# Study on Non-Destructive Testing Method of Existing Asphalt Pavement Based on the Principle of Geostatistics

**DOI:** 10.3390/ma18081848

**Published:** 2025-04-17

**Authors:** Duanyi Wang, Chuanxi Luo, Meng Fu, Wenting Zhang, Wenjie Xie

**Affiliations:** 1 School of Civil Engineering and Transportation, South China University of Technology, Guangzhou 510000, China; 2 Xiaoning Institute of Roadway Engineering, Guangzhou 510000, China; 3 Guangdong Communication Planning & Design Institute Group Co., Ltd., Guangzhou 510000, China

**Keywords:** 3D GPR, FWD, geostatistics, deflection, crack rate

## Abstract

In the context of the rapid advancement of reconstruction and expansion projects, there has been a significant increase in the demand for the inspection and evaluation of existing asphalt pavements. In order to enhance the efficiency and effectiveness of joint detection using 3D ground-penetrating radar and falling weight deflectometers, this study investigates non-destructive testing methods for existing asphalt pavements based on geostatistical correlation principles. The relationship between crack rate and deflection is analyzed using group average values. The characteristic sections division method based on the crack rate guideline was realized. Research on the prediction method for deflection using Kriging interpolation has been conducted. Research has revealed that there is a positive correlation between the crack rate and the deflection index. The principle of the singularity index can be employed to divide characteristic sections. The falling weight deflectometer is capable of conducting targeted testing in accordance with characteristic sections. Furthermore, the superior performance of Kriging interpolation in predicting deflection compared with linear interpolation has been demonstrated. According to the Kriging interpolation principle, the detection interval of slow lane deflection should not be more than 100 m. Kriging interpolation on one way lane of matrix data has the best effect, and it can predict deflection using a limited amount of slow lane and hard shoulder data. This facilitates analysis of the changing trend of the deflection index in cases where detection conditions are constrained. This method is of great significance for grasping the true performance status of the existing asphalt pavement structure.

## 1. Introduction

As the number of reconstruction and expansion projects continues to increase, there is an increasing demand for the detection and assessment of in-service asphalt pavement. Non-destructive testing (NDT) techniques are being utilized extensively due to their rapid detection speed and minimal damage to the pavement [1,2]. The most commonly employed techniques are 3D ground-penetrating radar (3D GPR) and the falling weight deflectometer (FWD). The 3D GPR employs electromagnetic wave reflection signals to detect internal damage within the pavement structure, enabling the distinct identification of disorders such as cracks, looseness, and voids in the pavement structure [3,4,5]. The FWD applies an impact load to test the overall strength of the road surface structure. The combined application of these two devices has been demonstrated to enhance the detection of existing problems in the pavement structure. However, several challenges have emerged during large-scale implementation, namely the discrepancy between spatial coverage and resolution. While 3D GPR can perform continuous scanning to acquire three-dimensional images, the FWD relies on fixed-point measurements. This discrepancy in the spatial sampling density may result in localised defect oversights. Secondly, FWD requires lane closure and is highly sensitive to road smoothness; in contrast, 3D GPR can perform detection without disrupting traffic. When conducting joint inspections, the time required to close the lanes must be balanced against the need for precision, which increases the complexity of the project.

3D GPR and FWD have been extensively utilized in the NDT evaluation of existing asphalt pavements. The Indiana Department of Transportation has utilized a systematic approach, incorporating FWD testing and 3D GPR data collection, to acquire critical insights into pavement performance characteristics. These characteristics are instrumental in determining project priorities and developing budgetary estimates for maintenance initiatives [6]. In the evaluation of flexible pavement, the 3D GPR is utilized to accurately measure the thickness of pavement structural layers and detect potential defects such as voids, delamination, and cracks. The FWD is employed to evaluate the overall load-bearing capacity and structural integrity of the road surface. The integration of the structural layer thickness data obtained from 3D GPR with the load-bearing capacity data from FWD facilitates a more comprehensive and precise assessment of the road surface condition [7,8]. The researchers utilized a 3D GPR to detect pavement thickness, and the FWD modulus inversion program employed the detected pavement thickness to enhance the accuracy of the results [6,8]. The FWD was utilized to analyze the bond between pavement layers and the strength of the structural layers, while the 3D GPR was employed to detect the type and size of the disease inside the pavement structure. The two devices were used in combination to assess the health of the pavement from different perspectives [7].

With the development of geostatistics, the statistical methods of geostatistics are gradually applied across the border in the civil engineering industry. The core of geostatistics lies in the modeling and prediction of spatially dependent data through methods such as spatial interpolation (Kriging), semi-variational function analysis, and spatial autocorrelation analysis [9]. In the field of tunnelling, Kriging interpolation plays a pivotal role in predicting and simulating grid models for various parameters. This ensures the reliability and representativeness of the tunnel design, thereby enhancing the efficiency and accuracy of the design process [10,11]. In geotechnical engineering, parameters such as soil bearing capacity, soil density and permeability coefficient are predicted using the Kriging method and semi-variational function method, especially in engineering design under complex geological conditions. The spatial variability of geomechanical parameters is analyzed through geostatistics, which effectively improves the reliability and validity of the indicators [12,13,14,15]. In the domain of structural engineering, the semi-variational function has been demonstrated to possess the capacity to quantify the spatial aggregation of crack length and density, thereby providing a quantitative foundation for the development of maintenance strategies [16,17,18]. In relation to materials, studies have demonstrated that by analyzing the spatial distribution of concrete surface roughness through the semi-variational function, the bonding performance between concrete layers of different ages can be predicted in order to optimise the construction process [19]. Furthermore, when analyzing the compressive strength of asphalt mixtures, the semi-variational function describes the spatial variation of the material’s internal porosity or strength gradient, which can then be used to assess the structural reliability [19]. Geostatistics is the scientific discipline that studies the spatial variation and spatial structure of natural phenomena based on the theory of regionalized variables characterized by spatial distribution [20,21,22]. Conventional statistical research is confined to the imputation of data, disregarding the spatial distribution of the sample. The research object must be a purely random variable, capable of repetition without limit, and the samples must be independent of each other. It should be noted that the geologic variables will vary depending on the geologic setting and are spatial. This methodological approach is particularly well-suited to the inference of spatial distribution in scenarios where data is limited, such as in the context of pavement inspection, where the health of an entire pavement is predicted from data obtained at a limited number of sampling points. However, at this stage, there are relatively few studies on the cross-border application of geostatistics and NDT of asphalt pavements.

With the increase of reconstruction and expansion projects, how to utilize existing asphalt pavement structure is a great issue faced by engineers and technicians, and how to efficiently and scientifically evaluate the service state of existing asphalt pavement is a problem that researchers must face directly. However, due to systematic differences, data density differences and other issues, researchers more often carry out joint analyses of independent data, and do not form good correlation relationships in the detection process. This paper utilizes the principle of geostatistics to carry out research on existing pavement inspection and evaluation methods based on 3D GPR guidelines, focusing on realizing the interactive inspection of 3D GPR and FWD during the inspection process, which improves the inspection efficiency, reduces the influence of the difference in data density between the two, and provides a new idea for the joint inspection of 3D GPR and FWD.

## 2. Research Methodology

In this paper, the Guangyun Expressway and the Yangmao Expressway in the Guangdong Province of China are used as the objects of analysis. Three-dimensional GPR is a 21-channel ground-coupled ground-penetrating radar system produced by 3D-Radar in Oslo, Norway. FWD is the Phonix PRI2100 system produced by Carl Bro in Grontmij, Denmark. The 3D GPR is used to detect the cracks of each structural layer and form the crack rate index of each layer, which is calculated as shown in Equation (1). The FWD is used for the detection of deflection index. Since deflection detection requires the use of point measurement, which is often time-consuming and requires the closure of traffic, the detection efficiency is very low; however, the crack rate can be obtained by 3D GPR detection, which does not require the closure of traffic and is more efficient [23,24,25]. Therefore, this paper proposes to utilize 3D GPR inspection to guide the characteristic road sections according to the crack rate index, then carry out targeted deflection strengthening inspections for the characteristic road sections, and finally extend the deflection inspection results through the principle of geostatistics to enhance the efficiency and effectiveness of the inspection of the in-service pavement structure of the reconstruction and expansion project.

(1) $$\gamma =\frac{l_{c}}{Area}\times 100$$

where γ is the crack rate, unit m/100 m^2^; $l_{c}$ is the length of the structural layer cracks in the section, unit m; and Area is the section area, with a section size of 50 m × 3.75 m, unit m^2^.

Firstly, an exploration and analysis of the relationship between crack rate and deflection of asphalt pavement under real traffic volume was conducted. In order to address the effect of spatial density inconsistency, the analysis method of grouped mean in geostatistics was adopted to study the correspondence. Subsequently, the principle of singularity was utilized to analyze the singularity of the crack rate index, and the segmentation method based on the crack rate index was investigated to identify the characteristic road sections with high and low crack rates. The subsequent stage of the research was to carry out the research of deflection detection method based on Kriging interpolation. This involved analyzing the effect of Kriging interpolation of different dimensions of deflection data and the influence factors of the interpolation effect. In addition, the reasonable spacing of deflection detection by the principle of semi-variable function was analyzed. Finally, the NDT method of existing asphalt pavement based on 3D GPR guidelines was constructed.

## 3. Basic Relationship Between Cracking Rate and Deflection

**Figure 1:**
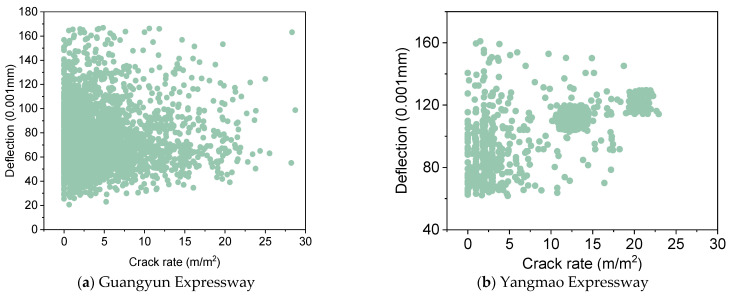
Corresponding patterns of crack rate and deflection.

The deflection index is a measure of the overall strength of the pavement structure, while the crack rate index of a single structural layer is indicative of the cracking state of that layer. To analyze the relationship between the deflection index and the crack rate index, the surface crack rate of the pavement surface, the crack rate of the bottom of the asphalt structural layer, and the bottom of the cement-stabilized gravel layer are accumulated to form the overall crack rate of the pavement structure, which is calculated as shown in Equation (2). The overall cracking rate and deflection correspondence pattern are illustrated in Figure 1.

(2) $$\gamma_{o}=\gamma_{s}+\gamma_{a}+\gamma_{c}$$

where $\gamma_{o}$ is the overall crack rate of the pavement structure; $\gamma_{s}$ is the surface crack rate of the pavement surface; $\gamma_{a}$ is the crack rate of the bottom of the asphalt structural layer; and $\gamma_{c}$ is the bottom of the cement-stabilized gravel layer.

**Figure 2:**
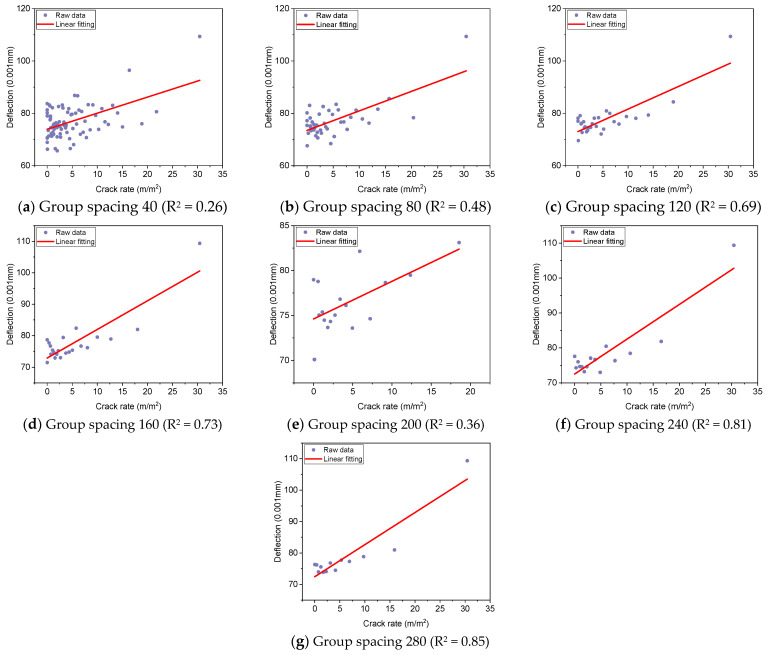
Crack rate and deflection corresponding form—arithmetic mean value (Guangyun Expressway).

In accordance with the principles of damage mechanics, it is posited that an increase in crack rate corresponds to an increase in deflection. However, as illustrated in Figure 1, a clear correlation between these variables is not evident. The fundamental reason for this discrepancy lies in the nature of the indicators themselves. The crack rate is a regional indicator, whereas the deflection is a point indicator. In the same region, the crack rate indicator remains constant, while the deflection indicator undergoes changes when the detection location is altered. Consequently, the conventional modeling method is unable to effectively establish a model relationship between the crack rate and the deflection. In order to further analyze the correspondence between the crack rate and the deflection, the crack rate index is ranked. Geostatistical data analysis methods are used to calculate the average crack rate and the corresponding average deflection at different intervals. The correspondence between the two is characterized by analyzing the relationship between the average crack rate and the average deflection. It is imperative to acknowledge that disparate grouping spacings will invariably give rise to disparate relationship models. In order to further analyze the model effects established by different grouping spacings, model analysis was performed for a variety of grouping spacings. The results are shown in Figure 2.

**Figure 3:**
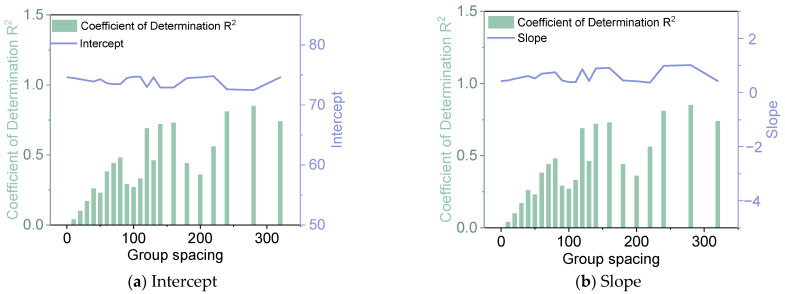
Fitting results of crack rate and deflection grouping (Guangyun Expressway).

**Figure 4:**
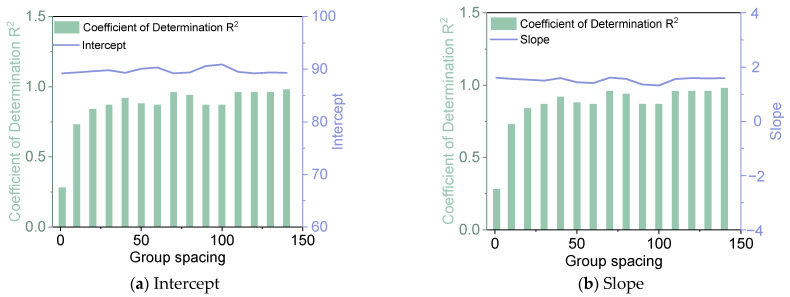
Fitting results of crack rate and deflection grouping (Yangmao Expressway).

As illustrated in Figure 2, with the increase of the group spacing, R^2^ values tend to increase. An evident correlation between crack rate and deflection emerges as group spacing is increased. The data demonstrate a positive relationship between deflection and crack rate, exhibiting a notable linear trend. Consequently, a linear model was employed for the fitting analysis, with multiples of 10 designated as the group spacing. The analysis was conducted for various group spacings, and the results are presented in Figure 3 and Figure 4 and Table 1.

As illustrated in Figure 3 and Table 1, the intercept indicators of different subgroups demonstrate a high degree of similarity, with an average intercept value of 74 and a coefficient of variation of only 1%. However, the slope indicators demonstrate significant variations, with an average slope value of 0.60 and a coefficient of variation as high as 35%. The average value of the coefficient of determination R^2^ is 0.48, and the coefficient of variation is 58%. The coefficient of determination R^2^ indicator reveals that the fitting results vary considerably, and as a whole, they tend to increase with the increase of the subgroup spacing. According to the fitting results, although there is a large variation in the slope, it can still reflect the positive correlation between crack rate and deflection, with the higher the crack rate, the greater the deflection. According to the fitting results of the coefficient of determination R^2^, the data fitting effect is optimal when the group spacing is 280, the intercept is 72.5 and the slope is 1.02.

As can be seen from Figure 4 and Table 1, there are some differences between the fitting results of the Yangmao Expressway and the Guangyun Expressway. The fitting results of Yangmao Expressway are relatively stable, and its crack rate is also positively correlated with deflection. The data fitting effect is optimal when the group spacing is 140, the intercept is 89.3 and the slope is 1.60.

In summary, the correlation between the deflection and crack rate indicators of the two roads is positive, and the higher the crack rate, the higher the deflection. The slope of the Guangyun Expressway is 1.02, while the slope of Yangmao Expressway is 1.60, which is quite different. The main reason for this difference is that the pavement structures are different. The pavement structure of the Guangyun Expressway consists of a 22 cm asphalt structure layer and a 56 cm cement-stabilized gravel structure layer, while the pavement structure of the Yangmao Expressway consists of a 15 cm asphalt structure layer and a 56 cm cement-stabilized gravel structure layer. Both are typical semi-rigid asphalt pavement structures. Due to the subtropical region, low temperatures are relatively infrequent, and cracking of the pavement structure occurs mainly in the semi-rigid base layer during operation. Since the Guangyun Expressway has a thick asphalt structural layer, if the cement-stabilized gravel layer cracks, the strength of the road structure is more likely to be maintained by the thick asphalt structural layer. However, the strength of the road structure with a thin asphalt structural layer is more likely to be affected by the cracking of the cement-stabilized graded aggregate layer. Therefore, there will be some difference in the slopes of the fitting models of the two roads. However, it is clear that the cracking rate is positively correlated with the deflection index. Therefore, the statistical analysis of the crack rate can be used to characterize the deflection.

## 4. Characteristic Section Division Based on Crack Rate Guidelines

### 4.1. Singularity Analysis Algorithm

The basic principle of singularity was first proposed by Professor Cheng Qiuming during his geochemical research, which defined singularity as a phenomenon involving a huge release of energy or a large amount of matter formation in a very small time-space region [26,27,28,29]. The traditional density-area fractal model is given by Equation (3) [28,30].

(3) $$\rho (\varepsilon )≃c\varepsilon^{\alpha -1}$$

where ρ(ε) is the density parameter; α is the singularity index, which characterizes the degree of measure singularity; and c is the corresponding local coefficient.

Positive singularity (α<1) indicates that the cracking rate of the road section is relatively high; negative singularity (α>1) indicates that the cracking rate of the road section is relatively low; and no singularity (α≈1) indicates that the cracking rate of the road section is relatively stable. The smaller the singularity index, the stronger the positive singularity, which can be used to directly spatially locate local abnormal areas and thereby identify the abnormality. The specific algorithm process is as follows:

(1) For any given spatial location, define the shape of the spatial window, with the window size increasing in sequence, such as $\varepsilon_{\min}=\varepsilon_{1}<\varepsilon_{2}<\cdots <\varepsilon_{n}=\varepsilon_{\max}$;
(2) Calculate the average crack rate $\rho (\varepsilon_{i})$ in each window;
(3) In double logarithmic coordinates, take $\log(\varepsilon_{i})$ as the horizontal axis and $\log\rho (\varepsilon_{i})$ as the vertical axis, and perform a linear fit to the data to estimate the slope α−1 of the linear model;
(4) repeating steps (1)–(3) by moving the window series to other positions to obtain a map of the spatial distribution of the local singularity index.

### 4.2. Singularity Index Analysis

**Figure 5:**
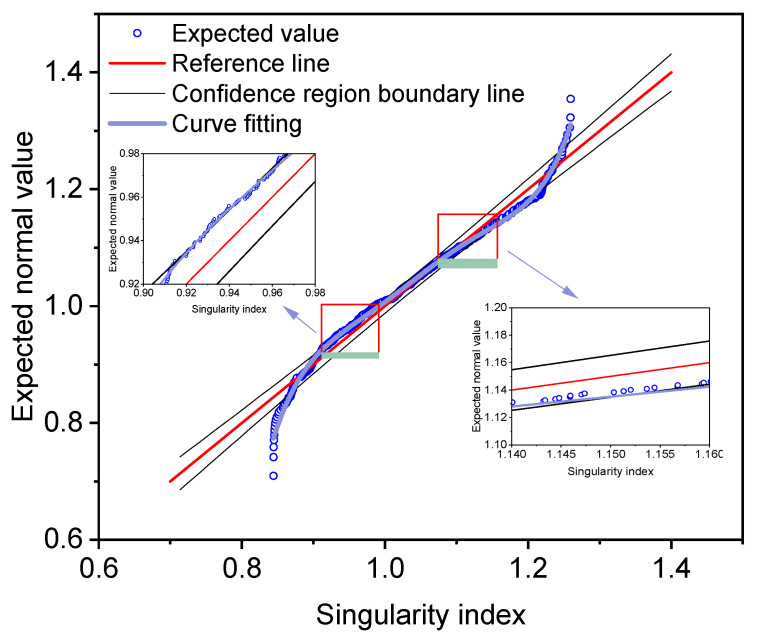
Singularity index analysis.

Based on the distribution pattern of singularity index in frequency, relevant researchers have proposed a singularity quantile analysis method for separating weak geochemical anomalies [29]. This method fits a normal distribution line, then sets a confidence interval for the residuals, and solves for the intersection points $\alpha_{1}$ and $\alpha_{2}$ of the singularity index fitting curve and the confidence interval line to achieve anomaly threshold segmentation. This article utilizes a 99% confidence interval through the normal distribution to restrict the range of the singularity index. An additional polynomial curve is fitted by the singularity index, as demonstrated in Figure 5, represented in blue. Ultimately, the intersection point of the fitted curve with the confidence interval is resolved to ascertain the breakpoint of the singularity index, thereby identifying anomalous data.

**Figure 6:**
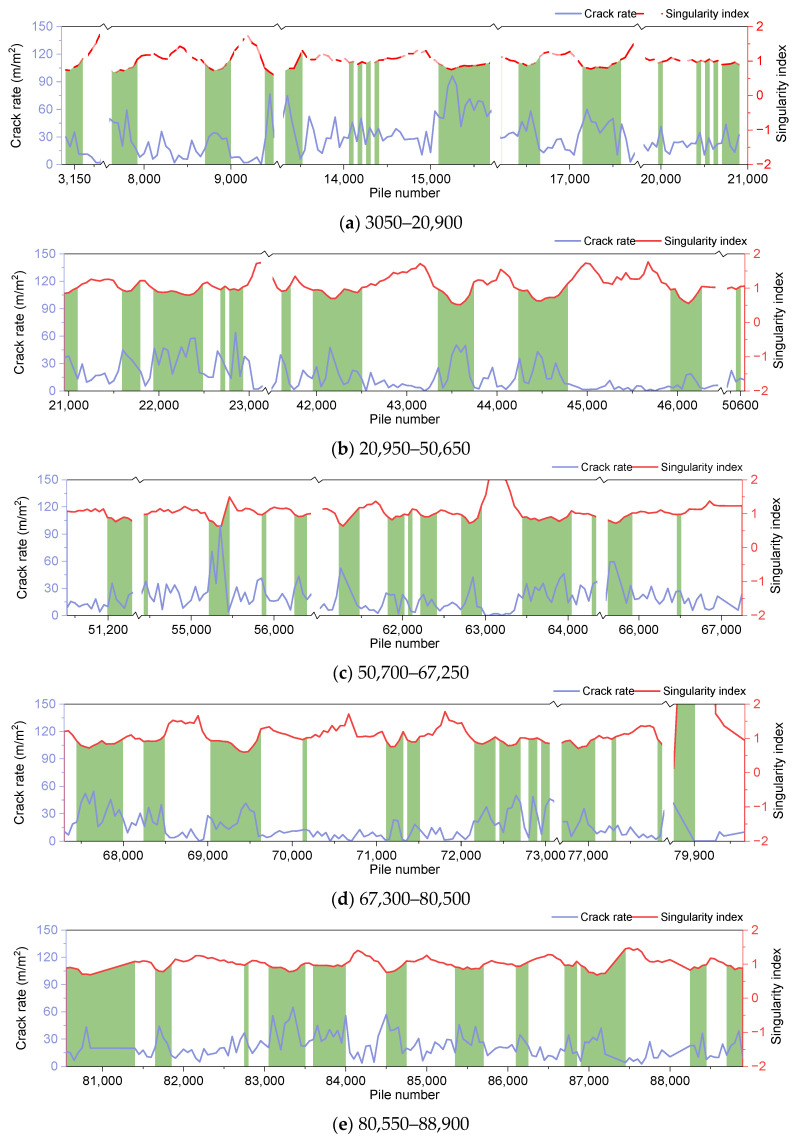
Corresponding relationship between the crack rate and the singularity index.

As illustrated in Figure 5, the intersection point solution indicates that when α > 1.15 or α < 0.94, the data is considered abnormal, exhibiting either a low crack rate or a high crack rate. To elucidate the impact of the singularity index, the singularity index and crack rate data are plotted for analysis, as depicted in Figure 6. To identify sections exhibiting a high crack rate, the data set with α < 0.94 is highlighted in green. As demonstrated in Figure 6, the singularity index is more effective at identifying data points with a high crack rate. However, the distribution of these points is too diffuse, making it impractical to test all of the data for deflection, as this would require the interruption of traffic in the lane. Consequently, it is essential to further segment the characteristic data and select the most indicative segments based on the prevailing conditions.

### 4.3. Characteristic Sections Division

**Figure 7:**
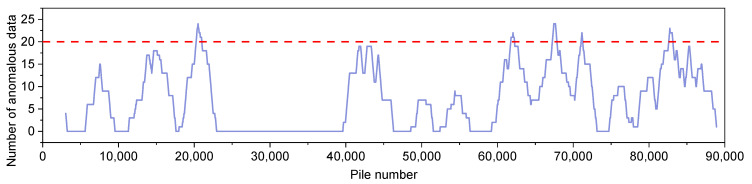
Number of abnormal data (the statistical area starts at the abscissa pile number and extends in the direction of increasing pile number for 2 km).

Given that the maximum length of traffic control on highways is generally limited to 2 km, the length of the detection area is set to 2 km. The number of abnormal data in the detection area is calculated, and the calculation results are shown in Figure 7. Since the length of the crack rate calculation area is 50 m, there are a total of 40 crack rate indicators in the detection area. When the detection area contains more than 20 (50%) abnormal data, the road section is designated as a characteristic section. As can be seen in Figure 7, 20,400–22,400, 62,050–64,050, 67,450–69,450, 71,150–73,150 and 82,750–84,750 are sections with high crack rates. Similarly, the sections with lower crack rates were analyzed and determined to be sections 13,750–15,700 and 43,750–45,750.

## 5. Deflection Detection Method Based on Kriging Interpolation

### 5.1. Analysis of the Interpolation Effect of Deflection Data in Different Dimensions

Kriging interpolation is a method based on the theory of variogram and structural analysis, which provides a linear unbiased optimal estimate of the values of regionalized variables within a finite region [31,32,33]. In order to analyze the interpolation effect of different dimensional deflection data, the difference in effect between Kriging interpolation and linear interpolation is compared and analyzed for data from a single-lane, one-way lanes, and two-way lanes.

(1) Single-lane data interpolation analysis

**Figure 8:**
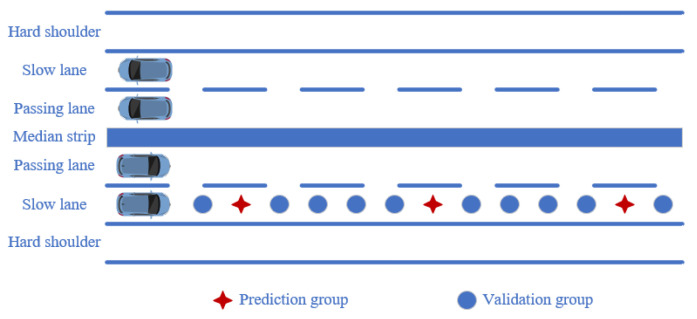
Schematic diagram for analysis of single-lane data.

**Figure 9:**
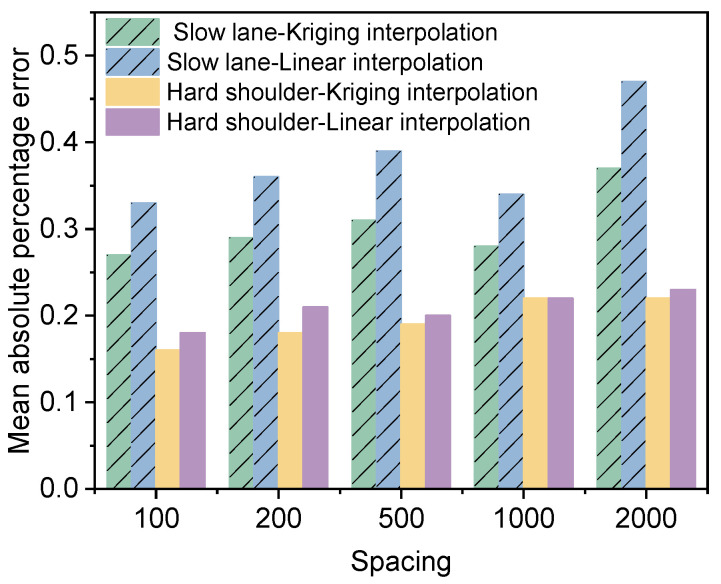
Effect of interpolation of one-lane data.

**Figure 10:**
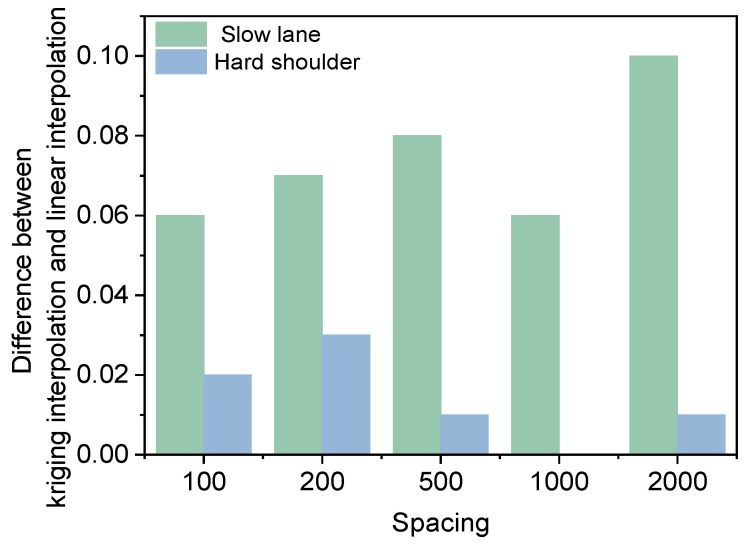
Difference between mean absolute percentage error of Kriging interpolation and linear interpolation.

The data in the slow lane and the hard shoulder are used to make interpolation predictions, and the differences in the effects of Kriging interpolation and linear interpolation are compared. The spacing between the data points for the deflection data is 50 m. In order to analyze the prediction effects of Kriging interpolation at different spacings, the data is filtered into five prediction groups with spacings of 100 m, 200 m, 500 m, 1000 m, and 2000 m, and the remaining data is used as the validation group. The schematic diagram is shown in Figure 8. A prediction model is established using the prediction group, and interpolation prediction is performed on the data in the validation group. The interpolation effect of the two different methods is analyzed by comparing the difference between the actual data and the predicted data in the validation group. The results are shown in Figure 9 and Figure 10.

As demonstrated in Figure 9, there are substantial discrepancies in the mean absolute percentage error of interpolation results in different lanes. The mean absolute percentage error of interpolation in the slow lane is notably higher than that in the hard shoulder, primarily due to the significantly higher coefficient of variation of the deflection in the slow lane compared to the hard shoulder. When data exhibits substantial variation, attaining a satisfactory interpolation outcome becomes challenging. The mean absolute percentage error of Kriging interpolation is consistently smaller than that of linear interpolation, thereby substantiating the superiority of the Kriging interpolation method. As the spacing of the data in the prediction group increases, the mean absolute percentage error of both Kriging and linear interpolation exhibit a tendency to increase. As illustrated in Figure 10, the difference between the mean absolute percentage error of Kriging interpolation and linear interpolation in the slow lane is greater than that in the hard shoulder, suggesting that the Kriging interpolation method may be more effective than the linear interpolation method when the sample data exhibits high variability.

(2) Multi-lane data interpolation analysis

**Figure 11:**
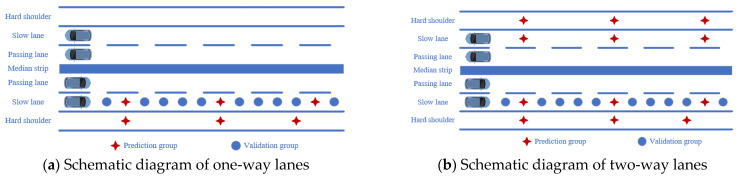
Schematic diagram of data of different dimensions.

**Figure 12:**
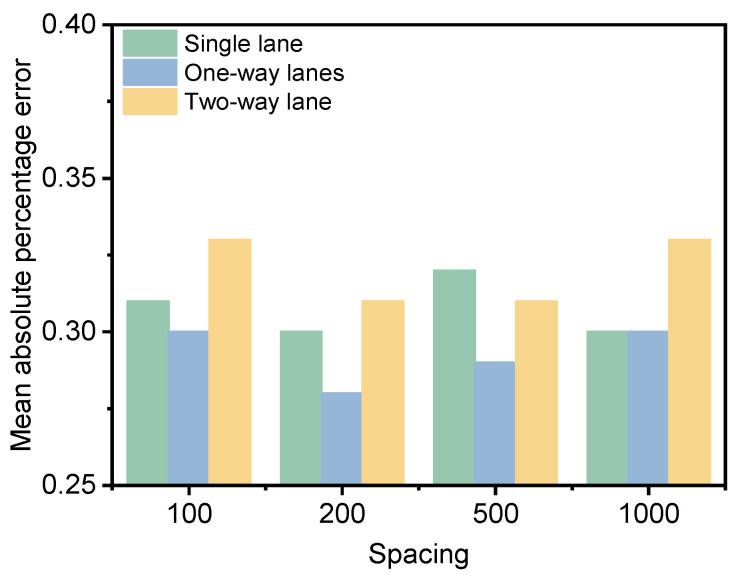
Interpolation results for data with different dimensions.

In order to further analyze the effect of Kriging interpolation of multi-lane data, the data of the slow lane and hard shoulder are statistically analyzed. Due to certain deviations in the data correspondence during the actual detection process, in order to further reduce the impact of the correspondence, only the data from 63,000–83,000 are used for the analysis of multi-lane data. To facilitate a comprehensive comparison of the effects of analyzing data from a single-lane, one-way lanes, and two-way lanes, the data was filtered into four prediction groups with intervals of 100 m, 200 m, 500 m, and 1000 m. The remaining data from the slow lane on the right were designated as the verification group. The schematic diagram is shown in Figure 11. The results of the interpolation are illustrated in Figure 12.

As can be seen from Figure 12, the Kriging interpolation effect is optimal for one-way lanes. The main reason for this is that the pavement structure layer of one-way lanes is constructed at the same time, and the loading effects of different lanes are relatively stable. The deflection indicators of different lanes have a relatively stable relationship and are relatively predictable. In addition, the predictive effect of the data matrix is inevitably better than that of a single lane, but the construction time of the road surface structure of a two-way lane is different, and the initial damage is inevitably inconsistent. The spatial relationship of the data is relatively weak, so using two-way lane data for Kriging interpolation is not the best choice.

### 5.2. The Impact of Data Variability on Kriging Interpolation

**Figure 13:**
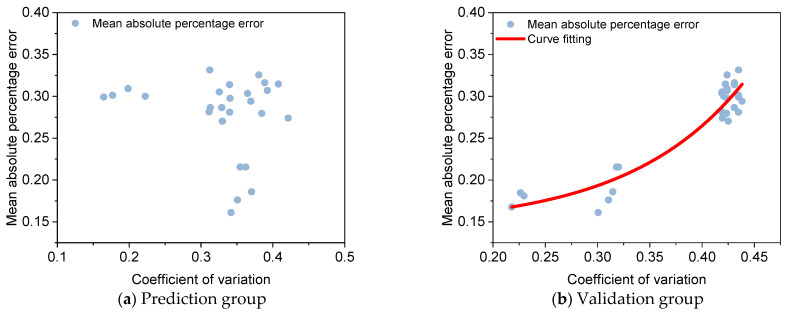
Coefficient of variation and Kriging interpolation mean absolute percentage error.

There is a significant difference in the mean absolute percentage error of the interpolation of the slow lane, hard shoulder, one-way lane and two-way lane Kriging. Even if the distance of the predicted group data is 100 m, the mean absolute percentage error of the slow lane interpolation reaches 0.26. However, the mean absolute percentage error of the hard shoulder interpolation is only 0.16, and there is a significant difference in the interpolation effect between the slow lane and the hard shoulder. In order to further analyze the main reasons for the differences, the coefficients of variation of the prediction group, the coefficients of variation of the validation group and the mean absolute percentage error of the interpolation results were analyzed in correspondence. The results are presented in Figure 13.

As can be seen from Figure 13, the coefficient of variation of the prediction group’s data has no direct relationship with the effect of Kriging interpolation, but there is an obvious non-linear relationship between the coefficient of variation of the validation group and the effect of Kriging interpolation; the fitting model is shown in Equation (4), and the coefficient of determination R^2^ reaches 0.89. In summary, it can be seen that the effect of Kriging interpolation and the variability phenomenon of the data itself are closely related. The Kriging interpolation method cannot predict the sudden variation phenomenon, but for continuous data, the variation can be effectively predicted. If the data itself varies more, the effect of Kriging interpolation will be greatly reduced.

(4) $$\psi =0.00324\;e^{\frac{Χ}{0.111}}+0.145$$

where ψ is the mean absolute percentage error of Kriging interpolation; Χ is the coefficient of variation of the validation group data.

### 5.3. Detection Spacing Analysis Based on Kriging Interpolation

**Figure 14:**
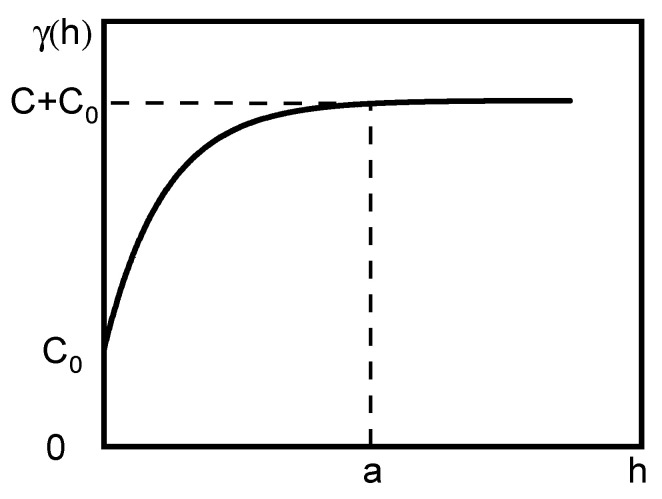
Semi-variogram model ($C_{0}$ is the block gold value; $C_{0}+C$ is the abutment value; C is the partial abutment value; a is the variable range).

The detection spacing that is too long may not reflect the deflection trend, while detection spacing that is too short will inevitably increase the test time. Therefore, how to reasonably determine the detection spacing for deflection is a key issue in the evaluation of old roads. Kriging interpolation minimises the prediction error mainly by fitting a semi-variogram function. The semi-variogram function is a continuous function used to describe spatial variation, reflecting the changing relationship between indicators and observations at different distances [34,35,36,37]. The specific formula is given in Equation (5), and a schematic diagram of the model is shown in Figure 14. Variable range (a) is the abscissa value when the value of the semi-variation function stabilises. If the detection spacing is within the variable range, the smaller the distance, the greater the spatial autocorrelation, and the greater the accuracy of the interpolation; if the detection spacing is greater than the variance, the regionalisation variable has no spatial autocorrelation, and the accuracy of the interpolation cannot be guaranteed. Therefore, the basic principle of interpolation can be used to determine a reasonable detection distance for deflection. The results of the semi-variogram fit to the deflection data on different expressways are shown in Table 2.

(5) $$r(h)=\frac{1}{2N\left(h\right)}\sum_{i=1}^{N(h)}{\left[Z\left(X_{i}\right)-Z\left(X_{i}+h\right)\right]}^{2}$$

where r(h) is the semi-variogram function; h is the distance between sample points in the spatial domain, known as the step size; $N\left(h\right)$ is the number of sample points at a distance of h; and $Z\left(X_{i}\right)$ and $Z\left(X_{i}+h\right)$ are the measured values of the regionalized variable $Z\left(X\right)$ at the spatial positions $X_{i}$ and $X_{i}+h$, respectively.

Table 2 shows that the variable range of the slow lane, which is subjected to vehicle loads, is relatively small, while the variable range of the hard shoulder, which is not subjected to vehicle loads, is relatively large. According to the above statistical results, when performing deflection detection, if the aim is to obtain detailed deflection changes, the sampling interval for the deflection of the slow lane should not be greater than 100 m, and the sampling interval for the deflection of the hard shoulder should not be greater than 1400 m. Deflection data within the above intervals can be obtained by Kriging interpolation to further improve the efficiency of obtaining deflection data.

## 6. Non-Destructive Testing Method Based on the Principle of Geostatistics

In the case of expressways that are open to traffic, it is inevitable that deflection measurements on the slow lane will require lane closures. For reasons of safety, the traffic management department permits a one-time lane closure of 2 km, which consequently results in an inefficient testing process. However, for hard shoulders that are not open to traffic, testing can be carried out with safety protection vehicles. When there is a high risk of interrupting lane traffic, 3D GPR can be used to detect crack rate without interrupting traffic and identify characteristic road sections based on the rate of cracks. Deflection detection is carried out on characteristic road sections of the slow lane, while fixed interval deflection detection is carried out on the hard shoulder. The Kriging interpolation method is used to predict deflection analysis for the entire road section, which greatly reduces the risk of interrupting traffic while improving detection efficiency.

**Figure 15:**
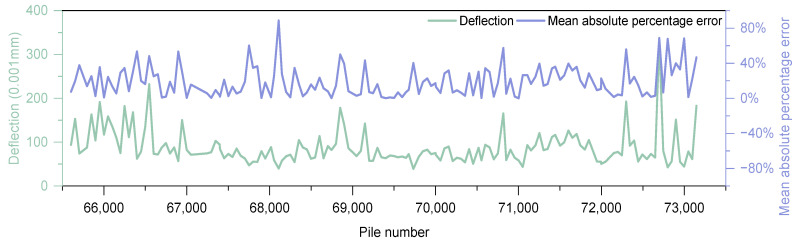
Validation group interpolation results.

The analysis section is 62,000–74,000. According to the above characteristic section division results, the slow lane selects the characteristic section 62,050–64,050 with a high crack rate as the deflection detection area, and the detection spacing is 50 m. In order to ensure the effectiveness of the slow lane Kriging interpolation, the entire hard shoulder is deflection detected with a spacing of 200 m. The above test data is used as the predicted group data. To verify the accuracy of the prediction results, deflection tests were carried out at intervals of 50 m along the remaining sections of the slow lane, and the test data was used as a verification group. The interpolation results are shown in Figure 15. The mean absolute error of the Kriging interpolation result is 22%. The interpolation result basically meets the needs of overall deflection analysis. As can be seen from Figure 15, the data points with large deviations in the Kriging interpolation results all appear in the area where the deflection results suddenly change, once again verifying the impact of the variability of the verification group data on the Kriging interpolation effect.

## 7. Conclusions

The NDT method based on geostatistics mainly uses 3D GPR to detect the crack rate of the pavement structure layer and determines the characteristic sections by the crack rate. Targeted deflection testing is carried out for the characteristic sections, and Kriging interpolation technology is used to predict the remaining deflection data. This method can quickly and efficiently obtain the trend of deflection index changes in the whole area when the testing conditions are limited; at the same time, the deflection data of the whole area can also be obtained by Kriging interpolation. The research process led to the following conclusions.

(1) A positive correlation has been observed between the crack rate and the deflection index; however, the relationship model for different road surface structures varies. It has been demonstrated that the thickness of the asphalt mixture structure layer has a direct impact on the deflection, with greater thickness leading to a reduction in the effect of the crack rate on deflection.
(2) The singularity index principle has been demonstrated to be an effective method for identifying the characteristic data of the crack rate. Furthermore, the data analysis method that utilizes fixed intervals has been shown to identify characteristic sections with high or low crack rates.
(3) Kriging interpolation is more effective than linear interpolation for predicting deflection data. The interpolation effect is limited by the variation of the data being interpolated. Kriging interpolation is most effective when the original data is one-way lanes matrix.
(4) When the detection spacing is maintained within the variable range index, Kriging interpolation can predict deflection with a limited amount of deflection data. This analysis can be used to examine the trend of deflection index changes when the detection conditions are limited.

Although the research and application of geostatistics have been carried out in this paper, there is no in-depth analysis on the interpolation prediction of deflection. The variation in the deflection value is also one of the reasons for the large deviation of Kriging interpolation. Further research is needed on how to further improve the effect of interpolation. The application of geostatistics principles to other non-destructive testing techniques is also the next step that needs to be expanded. At the same time, although the method of geostatistics is mainly applied to interpolation prediction, the application of the results after interpolation is not discussed, and the application of the data after interpolation can be further studied in the future.

## Figures and Tables

**Table 1:** Fitted effect summary.

Expressway	Parameter	Intercept	Slope	Coefficient of Determination R^2^
Guangyun	Mean	74.0	0.60	0.48
	Coefficient of variation	1%	35%	58%
Yangmao	Mean	89.7	1.52	0.9
	Coefficient of variation	1%	6%	19%

**Table 2:** Results of the fitting of the semi-variogram function to the deflection data.

Traffic Lane	Direction	Guangyun Expressway	Yangmao Expressway
Slow lane	Left	375	511
	Right	105	366
Hard shoulder	Left	2580	2668
	Right	1464	5590

## Data Availability

The original contributions presented in this study are included in the article. Further inquiries can be directed to the corresponding author.
